# Listerism and Another Step in Advance

**Published:** 1885-04

**Authors:** Alfred C. Girard

**Affiliations:** U. S. Army; Fort Porter, Buffalo, N. Y.


### BUFFALO

### Medical and Surgical Journal.

Vol. XXIV. APRIL, 1885. No. 9.

### Original Communications.

### Listerism and Another Step in Advance.

By Dr. Alfred C. Girard, U. S. Army.

Innovations have, at all times, had to contend with the opposition of fanaticism, ignorance, indolence or self-sufficiency. In past centuries reformers were burned at the stake; in the present age the printing press is a patient instrument for the same purpose and has been, and still is, extensively used in the employ of the enemy of progress. Our science has furnished its victims, and advancement has ever found ready opponents.

How many are there not who shrugged their shoulders when Pasteur first demonstrated the action of germs and Lister seized upon the idea to use it practically in the preservation of life? Pasteur has conquered, I believe, at least with those who take the trouble of investigating questions before expressing opinions. Lister, in spite of the marvelous results achieved by his antiseptic precautions, still has adversaries, and many authors in the literature of the day seem to delight in exhibiting cases apparently detrimental to his innovation.

Who are these enemies of Lister's system? First of all, those who are alike ignorant of its principles and of its benefits. Then, some who have pet theories which blind them to everything else; some, perhaps, who are jealous of Lister's fame; others, again (and for the honor of the profession be it said, probably the larger number), do not see any need for all these precautions; they have operated and treated successfully without them and obtained brilliant results.

They are "the Pharisees and Sadducees" of surgery, who thank God for their perfection. It is not to them that Lister has extended a helping hand. They believe they do not need it, and, therefore, despise it. But the "poor sinners," the hospitals which for centuries had harbored the sloughing wounds, the exhalations of fever, in short, everything noxious, abnormal, hurtful, that might be generated in the human organism,—the surgeons who had to operate under the most unfavorable circumstances, in the heat of summer, in crowded tenements, in epidemics, amid filth and poverty;—they welcomed this savior. Let the detractors of Lister and his doctrines go to those places where surgical success seemed to be impossible,—to those devoted German surgeons, who, in old buildings, in poorly supplied hospitals, amid dense populations were battling for their patients' lives against the dread trio, erysipelas, gangrene and pyæmia—and they will hear tales of woe of former times and of a new happy era consequent upon the introduction of Lister's wound treatment with an almost fabulous decrease of mortality rate. I have almost a sentiment of pity for the blindness and ignorance of Lister's adversaries, were it not that I believe that in the face of these overwhelming proofs they assume an awful responsibility in rejecting what, with some increased trouble to themselves, may prove a strong, if not absolute safe-guard for their patients. The day will come, even for the favorable conditions of surgery in this relatively new country, when our excellent sewerage and ventilating systems, our pure air and good water supply (theoretically!) will not be the protection they are now and when the favorable circumstances which seem to render unnecessary these surgical precautions, will make room to the results of over-crowding and contamination of buildings and then antisepsis will no more be a matter of choice, but a dire necessity, and the victims who have fallen here and there to the dread wound complications will cry to heaven for vengeance on those who have neglected to make their fate almost an impossible one. We cannot know where, in a palace or a hovel, these germs await us to destroy the healing results of our operations or lead injuries to a fatal result, and it is our duty to guard against such possibilities. Wise people, when cholera is known to be on the march, do not wait providing a pure water supply, perfect sewerage and removal of garbage until it has commenced claiming its victims in their town. Why should we surgeons, who are supposed to lead with good example, wait with our surgical precautions until the death list compels us.

The time for discussion of the usefulness of antisepsis is as much past as the question of the possibility of telephones, and the only war upon it worth considering is that made by men who over-estimate the danger of the means used or wish to see them entirely safe. Of course we do not propose to convince those who labor under the fallacy that Listerism means simply application of carbolic acid to wounds. Such ignorance was excusable some years ago. Still, for the benefit of those who have been remote from opportunities of information, I will state, in a few words, that the essence of Listerism is to prevent the entrance into wounds of germs floating in the atmosphere or adhering to dressings, instruments, fingers, etc., which may come in contact with these wounds. It has been shown by Pasteur that these germs bring about changes in animal fluids which eventually lead to putrefaction, and Lister, on the assumption which since has been proven to be correct in numberless instances, that they are responsible for the complications resulting in wounds and injuries, sought to prevent their entrance or contact with the discharges and to destroy them, if those conditions arise, showing that the precautions were not properly taken or entrance of germs had taken place at the time of injury.

For this purpose Lister used, at first, a carbolic paste, then the spray with carbolized water and hygroscopic gauze impregnated with the acid; but always mindful of the possible ill results of the absorption of the acid, he protected his wounds from direct contact with the germicide wherever possible. Later on, he or his disciples, used other agents, such as boracic and salicylic acid for the same purposes, and for the spray thymol and other ethereal oils inimical to the germs. It is, therefore, evident that Listerism is not necessarily identical with carbolic acid, nor a departure from the acid a departure from Listerism.

That there is a certain danger from the possible absorption of carbolic acid used in the ablution of the wounds is not denied, although such occurrences can still be counted among the hundred thousands of cases treated according to Lister.

As our knowledge of bacteriology advanced, we found and substituted other agents, more destructive to the germs and less dangerous to the human organism, but in whatever form they were used—in solutions, as the bichloride of mercury, or in powders, as the iodoform—they still, here and there, led to unpleasant results, without, however, impeding the accomplishment of the main object, asepsis. The most faithful followers of Lister have not been blind to these disadvantages, and have steadily tried to assist their master in the perfection of the technique; but some of the changes proposed were too complicated, others too expensive, others not effective enough, and the great desideratum has not been attained until very lately, when it appears that a method has been devised uniting all the safeguards of the Lister system with absolute safety to the patient. It is by substituting the oxide of zinc for all the preparations to be brought in contact with the wound, excepting when a septic condition of the wound necessitates the stronger agents originally employed by Lister.

The introduction of this new method is due to Professor A. Socin, of the University of Basle, my former teacher, whose eminent qualities I almost worshipped when a student twenty-five years ago, an appreciation which has not been lessened by the judgment of riper years and comparison with other coryphees of science. I know that implicit reliance can be placed in his logical reasoning and accuracy of statements, and I am, therefore, prepared heartily to endorse, when otherwise I might wait for corroboration.

I proposed at first to submit a condensed description of Socin's method, but I find it so admirably given in his preface to the annual report of the city hospital of Basle, and coupled with such other judicious advice, that in the interest of my readers I prefer giving a translation of the same.

"Since the commencement of 1883," he states, "the method of wound treatment in the surgical section under my charge has been considerably modified, and I propose to make a short report on the subject. Up to the time mentioned, during eleven full years, we had observed, with few exceptions, rigidly, Lister's precepts, and had adopted those changes and simplifications only which had been recommended by Lister himself. At the introduction of the powder dressings, particularly the iodoform, we made experiments with them, and became satisfied not only of the convenience and simplicity of the procedure, but also of its dangers. My friend, Dr. Picard, Professor of Chemistry, with whom I often discussed the question of antiseptis, drew my attention to the oxide of zinc; after a number of experiments in the culture oven, and practical trial, I found this substance as effective as iodoform and bismuth; besides this, however, the oxide of zinc has the very valuable qualities of being absolutely inocuous, entirely odorless and very cheap."

At the meeting of the central society of physicians which took place in Basle on May 25th and 26th, I was already enabled to report on the satisfactory results which we had obtained with the oxide of zinc. Soon after I read (*Deutsche Med.*

*Wochenschrift*, June, 1883) that Prof. Peterson, in Keil, had obtained very satisfactory results with the same agent. Our mode of application differing in many respects from that of Prof. Peterson, it will not be undesirable if I describe our method. The main idea guiding us is that no other antiseptic shall come in contact with the wound. We use, therefore, carbolized solutions only for the disinfection of the operating room before operation, to wash the hands, instruments and the field of operation. The sponges, after being washed clean in water, have to remain four weeks in a five per cent. solution of carbolic acid before being used again, and then washed out and laid into the weak zinc milk (see below). We use the oxide of zinc in four different forms:

(*a*) A mixture of one part oxide of zinc and one hundred parts distilled water (thin zinc milk) is employed for irrigation of fresh wounds, immersion of sponges which come in contact with the same, and for the purpose of washing out cavities of the body and joints, which have been opened.

(*b*) A mixture of ten parts zinc with one hundred parts distilled water (thick zinc milk) is used to irrigate all those wounds which are to remain open (wounds of cavities, wounds of operation in mouth, rectum, etc.) before dressing, until the whole surface is covered with a white film.

(*c*) In form of dry powder it is applied with the sprinkling box in ulcers, burns, scratches, etc., and in these it has had very favorable results.

(*d*) For the covering of wounds closed by suture we employ the so-called zinc paste, consisting of fifty parts zinc oxide, fifty parts water, and five to six parts chloride of zinc. This paste has the peculiarity of rapidly drying on exposure to the air and forming a solid scab, which is the more adherent and hard the greater the proportion of the chloride in the mixture. Both substances combine chemically to an oxychlorine, which is frequently used in dentistry as a cement. In the proportions mentioned above, containing the oxide largely in excess, it forms an air-tight, sufficiently adherent, wound covering, especially when a thin layer of cotton wadding is used as a support to the paste in its application with the brush. When wounds are drained it is self-evident that the opening of the drain must not be covered. Wounds which are not drained, but placed in exact coaptation by suture, do not require any further dressing after the application of the paste. This is particularly convenient in wounds of the hairy part of the scalp, wounds of the face, or in the neighborhood of orifices (harelip and other plastic operations). After eight to ten days this scab commences breaking off and finally falls of with the remainder of the unabsorbed catgut threads. The scars remaining are usually more delicate and perfect than any I have witnessed with any other wound treatment.

We use hygroscopic gauze for the purpose of covering and absorbing drained and open wounds, as it offers the best absorbing power of any dressing recommended. It is folded and well impregnated with the thin zinc milk, which is wrung out before applying the dressing. In this slightly humid condition its absorbing power is at its maximum, as evidenced by numerous experiments. It is then fastened with dry strips of the same material, which also serve the purpose of compression. A second layer of dressing is then added, composed of several sheets of wadding, or a peat cushion expressly prepared for each case. I believe the report herewith submitted offers evident proof that even large fresh wounds may be kept aseptic under the zinc dressing, and that one application may be sufficient. The method has, however, its drawbacks. The constant shaking of the mixture used for irrigation is especially inconvenient, and we are using, therefore, for that purpose, of late solutions of bichloride of mercury, although when largely applied they may easily cause stomatitis. The zinc oxide is as useless as the other insoluble antiseptics where the wounds are already septic. Here we use, as heretofore, solutions of corrosive sublimate, carbolic acid or chloride of zinc. I consider it self-evident that in antiseptic wound treatment the choice of the particular antiseptic or dressing material is of less consequence than a careful adaptation to each particular case, of the principles of the method. The most important among the few comparatively simple measures needed to carry out the same is that exact coaptation of the tissues be achieved when union per primam is attempted. It is clear that it is not sufficient to sew up the skin, however carefully it may be done, in a wound embracing several layers of structures. I believe, therefore, that any adherent of antiseptic surgery, will, of himself, adopt the deep sutures (*versenkte Nähte*) which have, of late, been recommended as a great novelty. We have been using them a long while already, under the name of *Etagennath.* An amputation wound, *e.g.*, will never heal fully, per primam, if we are satisfied with covering it with a flap of skin united by sutures at its edges. We have, therefore, entirely abandoned this formerly favorite mode of amputating and have returned to the old circular operation in two stages, or when the circumstances permit it, to the muscular flap. In the circular operation we first coaptate the deep muscles on either side of the sawed off bone with sutures to a linear union. This is followed by suture of the superficial muscles, then the fascia, and finally the skin. In the muscular flap the cut is so calculated as to allow a careful union of muscles from the deeper parts outwards. In this manner we not only avoid formation of a "dead space" in the wound, but may reduce drainage, this necessary evil, considerably. If in addition to this we follow the rule of the old French surgeons, of removing all the larger nerve trunks by resection from the wound, we shall see the stumps heal without suppuration or pain, and what is of greater importance yet to the patient, they will be more useful. The very inconvenient union of the bone and skin by the cicatrix is thus avoided, and artificial limbs can be easily adapted. The muscles united by suture have no tendency to retraction, and form an excellent permanent bolster, movable over the bone. I have satisfied myself that in stumps after exarticulation, the soft parts preserved as thick as possible and united per primam, do not adhere to the joint covered by them, but form an actual articulation, thus markedly increasing the usefulness of the stump. The wounds resulting from the removal of deeply seated tumors, operations on bones and opening of abscesses, should be closed on the same principle. In this manner the painful, deforming, adherent cicatrices may be avoided, in many cases."

Thus far the report of Prof. Socin, although, as mentioned above, the latter part of this report does not bear any direct relation to the question of antiseptic dressings, it contains such useful hints that I feel justified in digressing thus far.

The number of operations reported treated on antiseptic principles, and wherever suitable, with zinc, is 353, with fourteen deaths. These latter were the final termination of three cancerous tumors, five tracheotomies (four for diphtheria and one performed by the patient with suicidal intent), two incarcerated herniæ (in one from degeneration of liver and kidneys, the other gangrene of intestine), one resection of the hip (death from miliary tuberculosis of the lungs, kidneys and spleen), the remainder fatal lesions, *per se*, surely favorable results, as far as the wound treatment could bring them about. Of wound complications, which are supposed to be prevented by careful antisepsis, he reports two cases, both erysipelas, one following the scraping out of the knee-joint several months after resection (dressing of salicylated cotton), the other nineteen days after a severe railroad injury brought in already septic with a complicated fracture of the humerus. Origin from a counter-incision in axilla (open wound treatment) compresses of five per cent. carbolized solution. Both cases recovered in less than two weeks.

This oxide of zinc dressing is very easily applicable in private practice. No particular caution need be taken with the proportions; the zinc milk can be prepared at the bed-side; the number of dressings necessary are few, as a rule one is sufficient. When the powder form is used it can safely be entrusted to the patient or family, and does not betray itself by any odor. With some hydrophile gauze, a box of oxide, a little chloride, a bottle with cat gut and his pocket case the surgeon may feel equal to the requirements of most cases of wounds or operations performed in private practice, and these substances are surely a small price paid for the safety of the patient and the honor of a good result.

FORT PORTER, BUFFALO, N. Y.

